# The Institutional Worker

**Published:** 1919-11-08

**Authors:** 


### INSTITUTIONAL WORKER

**Being a Special Supplement to "The Hospital"**

### OUR BUREAU OF INFORMATION.

### Rules for Correspondents.

1. Every letter must be accompanied by the coupon to be cut from the back cover (inside page) of THE HOSPITAL, current issue, and must contain the name and address of the correspondent with pseudonym for publication if desired. Replies by post cannot be given save under exceptional circumstances at the Editor's discretion.

2. Letters from Approved Homes in reply to special needs published in the Bureau should state terms and full particulars, and be sent prepaid under cover to the Editor of the Bureau with name written across coupon for identification.

3. Proprietors of Homes which have not yet been entered on the List of Approved Homes, but have spare accommodation likely to suit special needs, are invited to write for an application form for registration. The fee for registration, which includes two announcements of the Home in the Bureau and other privileges, is 10s.

4. All communications to be addressed to the Editor of THE HOSPITAL, 28 Southampton Street, Strand, London, W.C. 2, and marked "Bureau of Information."

### INSTITUTION FACTS AND FIGURES.

### A Convalescent Home Time-Table.

#### Answer to September Question.

**By S. C. H.**

THE question was :

**Draw up a daily programme with suitable suggestions regarding exercise, light work, amusements, rest hours and diet to be adopted in a Convalescent Home for working girls situated in the country, and occupied mainly by severe cases of anæmia and debility. The ages of the girls vary from 13 to 30, and as the staff consists of matron and cook-general only, each patient, unless confined to bed, is expected to help with light household tasks. The average number of patients is 12. A plentiful supply of milk and eggs is available.**

WITH severe cases of anæmia and debility it is well to keep the newcomers in bed for the first two days after their arrival at the home, so that if the average number of patients is twelve, we may generally count on ten girls for the ordinary routine of exercise, work, rest, and play. This would also allow for an occasional day off on the part of any patient below par. We should suggest that for the purposes of the morning work at all events, the inmates of the home should be divided into parties of five girls in each. Each party should take it in turn to be downstairs at 8 A.M. for breakfast, which of course would be prepared and cooked by the cook-general. The five girls on duty should wait on the other seven and take breakfast upstairs to the bedrooms. Thus every alternate day each patient would have the advantage of a long rest in bed. After breakfast—from 8.30 to 9.30—the girls on duty might help with light work, such as dusting, washing-up, preparing vegetables, etc., for lunch, and of course all able to do so would make their own beds and keep the bedrooms in good order.

At 11 A.M. the matron should see all the girls, give directions (where such are needed) as to medicines and treatment, and some nourishment such as a beaten-up egg in a glass of milk should be given to each patient.

From 11 A.M. to 1.30 P.M. each patient strong enough should be out of doors. Even in wet weather it is important that there should be a short walk for the sake of the fresh air. Those in a very delicate state of health should not be allowed to walk much at first, but should be encouraged to sit outside if the weather is warm.

Dinner at 1.30 should be followed by rest for all until 4 P.M. Books and needlework might be permitted, but the rest should be as complete as possible and in the open air if the weather will permit. The girls who were not on morning duty should get the tea, and thus give an opportunity to the cook-general to be completely off duty. After tea—4.30 to 5 P.M.—dishes should be washed and supper laid, and if necessary prepared by the patients.

In summer the girls should again be outside and could participate in organised amusements in the open air. Supper at 8.30, so that all may be off to bed in good time. Lights out at 10 P.M.

The diet selected should be plentiful and nourishing, but palatable. It must be remembered that anæmic girls are often very fanciful, and as far as possible individual tastes should be studied—the aim being to restore the system by building up, for in these cases good food is even more important than tonics. With a good supply of milk and eggs at her disposal, the matron will find it easy to prepare nourishing and tasty dishes, and it should be ascertained that each patient takes a certain quantity of milk and at least one egg per day.

Fruit in season, particularly apples, prunes, figs, should form part of the menu; anæmic girls are particularly liable to constipation, which is often aggravated by iron tonics. Meat should not be overdone—raw-beef sandwiches may be required for the severe cases Most kinds of fish are helpful, and potatoes and green vegetables. In short, the patients can partake pretty well of normal diet—though indigestible and greasy foods, such as pastry, pickles, etc., should be avoided.

The bedding and beds should receive the matron's careful supervision. Girls suffering from anæmia and debility are generally cold mortals and require plenty of warm, light clothing. The beds should be of simple construction of the hospital variety.

As regards amusements, tennis, croquet, gardening, walking, quoits, and clock golf are suitable, and many other outdoor pursuits will suggest themselves. The more strenuous games, such as hockey, net-ball, and the "digging" part of gardening, should be avoided, as the patients are not fit for too much exertion.

For indoor games dancing (in moderation) Swedish drill, ping-pong,

(*Continued on p. 2, col. 3*).

### Suggested Time Table for Patients and Staff.

| | Morning | Afternoon | Evening |
|---|---|---|---|
| Patients (on duty)— | Breakfast at 8 A M : take meals to bed-patients. 8.30 to 10.30 light household tasks. 11.0 report to matron. 11.15 to 1.30 P.M., exercise in fresh air. 1.30, lunch. | Rest 1.30 A M. to 4.0 P.M. 5.0 to 6.0 light housework. | 6.0 to 8.30 P.M. in open air if fine. Indoor amusements if wet. Supper, 8 30; bed, 9.30; lights out, 10 0. |
| Patients (off duty)— | Rise at 10.30 A M. and report to matron at 11.0. Exercise in fresh air as above. | Rest 1.30 to 4.0 P.M. 4 0 P.M. prepare tea. | Same as patients on duty. |
| Cook-general— | Rise at 6 30 A M. On duty until 2.0 P.M. | Rest every day and off duty 2 0 to 6.0 P M two half days 2.0 to 10.0 per week as arranged with matron. | 6.0 to 9.0 P.M. on duty, except on half days. |
| Matron— | Rise at 7.0 A.M. | Off duty two half days per week when the cook is on duty. | 6 0 to 9.0 P.M., off duty. 10.0, inspection to see that all lights are off. |

### Question for November.

### Reducing Hours without Increasing Staff.

**State how you would deal with the problem of reducing the weekly hours of the Male Manual Staff of a Hospital from say 60 to 48 without increasing the number of such Employees. There are 6 Porters, 2 Boilermen, 1 Laundryman, 3 Gardeners (one of whom assists inside), and a Boy. The Hospital is built upon the pavilion plan, has long corridors (glass roofs and sides) and large grounds. All window-cleaning is done by the Porters. There are 2 steam and 4 hot-water boilers, the Heating System not being central. 187 beds.**

**(A minimum payment of 10s. 6d. will be made for each published answer.)**

RULES.

The following rules must be observed:—

1. Contributions must be written on one side of the paper. Conciseness and terseness are desirable features. The MS. must bear the name and address of the sender and be accompanied by coupon to be cut from the back cover (inside page) of the current issue of THE HOSPITAL. A pseudonym must be chosen if the name is not to be published.

2. Contributions must be addressed to the Editor of THE HOSPITAL Institutional Worker Supplement, 28 & 29 Southampton Street, Strand, London, W.C. 2, should reach him before the end of the current month, and be marked in the left-hand corner "Facts and Figures."

### QUESTIONS INVITED.

IN connection with our Question Box, we offer every month five shillings each for the best questions which are sent in for consideration. The questions may be on any institutional subject and concern any department, from the secretary's to the porter's, or the matron's to the domestic staff; the one essential is that they must be practical and deal with points that have a definite relation to hospital or institutional administration, upkeep, finance, or management. Points relating to artificers' work, housekeeping, the laundry, out-patients, and other practical matters will be welcome. It is hoped that thus, when difficult or doubtful points arise in the course of their work, institutional workers will be encouraged to put them in the form of a question and send them to the Editor, THE HOSPITAL Bureau of Information, 28 & 29 Southampton Street, Strand, London, W.C. 2, marked "Question Box."

The best questions will be published in due course and our readers will be given the opportunity of answering them. Thus all institutional workers may be able to co-operate with the view of helping the work and smoothing the difficulties of each other. In short, we want the Question Box of the *Institutional Worker* to be freely used by every worker habitually.

### ENQUIRIES AND ANSWERS.

### *THE SICK AND IN NEED.*

#### Home for Maternity Case.

WE do not recommend any home other than those approved by us. We have placed a note in these columns asking the homes in the neighbourhood you mention whether they can receive a maternity case. Housing difficulties since demobilisation have created an abnormal demand for accommodation of this character. We regret we cannot give postal replies; a coupon should be enclosed with each inquiry.—C. M. W.

### *EMPLOYMENT AND TRAINING.*

#### Training in Dundee.

SUITABLE applicants are received at the Dundee Royal Infirmary, after six months' trial, for three years' training; candidates must be between twenty-one and thirty-two years of age. There is a separate department for maternity nursing, and pupils are trained for the C.M.B. Apply to the Matron.—JESSIE.

#### Massage Training in Manchester.

THERE is a School of Massage attached to the Royal Infirmary, Manchester, and students are prepared for the examination of the Incorporated Society of Trained Masseuses. There is a Hostel for Students in connection with the School. The Ancoats Hospital also has a School of Massage and Medical Electricity. Application should be made to the Matron in each case.—M. E. M.

#### Hospital for Mental Case in Ireland.

YOU might write to the Secretary of the Hospital for Mental Diseases, Palmerston, Chapeligod, Co. Dublin, who will give you details of admission to this institution. English cases are received upon payment of not less than £60 per annum. An extra charge is made if special attention is required, also all clothes that may be provided are charged for.—IRISH.

#### Nurse for Poor Jewess.

WE think you will obtain the help you require through the Jewish Maternity District Nursing and Sick Room Helps' Society. This Society sends out nurses or sick-room helps to nurse poor Jewesses in their own homes, and has accommodation for six maternity cases in wards. We suggest that you write to the Superintendent, Nurses' Home, 24 Underwood Street, E. 1.—JEWESS.

#### Work for Disabled Men—Bookbinding.

There seems to be no training-centre under any educational scheme yet established which includes the craft of bookbinding. It is explained that there does not exist sufficient scope for progression after training. Many disabled soldiers are skilled bookbinders, and their individual services can be obtained by inquiring through the Special Employment Exchange, 6 Catharine Street, Strand, W.; the L.C.C. Educational Department, Ministry of Labour, Hamilton House, Temple Avenue, Victoria Embankment; or to Mr. W. J. Norrell, Member of the Consultative Committee for Bookbinding, 17 Dean Street, Soho.

### *MISCELLANEOUS.*

#### Hostel for Nurses.

THE hostel you refer to is, we think, Queen Mary's Hostel for Nurses, which has only recently been opened at 194 Queen's Gate, London, S.W. 7. There is also one in connection with this in Warwick Square, which gives a night's hospitality to nurses passing through London. Write to the Lady Superintendent at Queen's Gate.—NURSE G.

#### War Memorial for Military Nurses.

THE subscribers to the War Memorial for Military Nurses who have made the supreme sacrifice during the war is limited to past and present members of Queen Alexandra's Imperial Military Nursing Service and its Reserve, the Territorial Force Nursing Service, and assistant nurses and special military probationers attached to either service. As you are a demobilised member you will be required to send your contribution to Messrs. Holt and Co., 3 Whitehall Place, London, S.W.—T.F.N.S.

### *ACKNOWLEDGMENTS.*

#### Home Required for Maternity Case.

CAN any of our approved homes in the neighbourhood of Watford or Bushey receive a maternity case? Replies should be addressed "C. M. W.," c/o The Editor.

#### Convalescent Home Time-Table.

(*Continued from page 1.*)

and table games such as cards, draughts, and halma should be encouraged. The patients should have access to a fiction library, and might with advantage be taught some simple occupation such as basket-making, passe-partout work, embroidery, pen-painting, crotchet, etc., for it is a great point to keep the patient's mind and body fully occupied and so combat the depression which so frequently accompanies debility.

Once to church on Sunday is quite enough. It is better for the patients to be out of doors than to be shut up for half a day in a place of worship. If a simple service can be arranged in the home itself, so much the better, and this should, if possible, be held in the open air.

From the above remarks it will be seen that a suitable time-table might be drawn up on lines suggested on the previous page.

